# Analysis of human papillomavirus type 16 E4, E5 and L2 gene variations among women with cervical infection in Xinjiang, China

**DOI:** 10.1186/s12920-024-01926-3

**Published:** 2024-07-04

**Authors:** Haozheng Cheng, Yangliu Dong, Le Wang, Xian Zhao, Xiangyi Zhe, Dongmei Li, Hongtao Li, Renfu Shao, Jing Tuo, Zemin Pan

**Affiliations:** 1 Department of Biochemistry and Molecular Biology, School of Medicine, Xinjiang Endemic and Ethnic Disease and Education Ministry Key Laboratory, Shihezi University, Shihezi, 832002 Xinjiang China; 2 Centre for Bioinnovation, School of Science, Technology and Engineering, University of the Sunshine Coast, Maroochydore, 4556 Australia

**Keywords:** Cervical exfoliated cells, Human papillomavirus, Genetic polymorphism, Sanger sequencing

## Abstract

**Background:**

There is a high incidence of cervical cancer in Xinjiang. Genetic variation in human papillomavirus may increase its ability to invade, spread, and escape host immune response.

**Methods:**

HPV16 genome was sequenced for 90 positive samples of HPV16 infection. Sequences of the E4, E5 and L2 genes were analysed to reveal sequence variation of HPV16 in Xinjiang and the distribution of variation among the positive samples of HPV16 infection.

**Results:**

Eighty-one of the 90 samples of HPV16 infection showed variation in HPV16 E4 gene with 18 nucleotide variation sites, of which 8 sites were synonymous variations and 11 missense variations. 90 samples of HPV16 infection showed variation in HPV16 E5 and L2 genes with 16 nucleotide variation sites (6 synonymous, 11 missense variations) in the E5 gene and 100 nucleotide variation sites in L2 gene (37 synonymous, 67 missense variations). The frequency of HPV16 L2 gene missense variations G3377A, G3599A, G3703A, and G3757A was higher in the case groups than in the control groups.

**Conclusions:**

Phylogenetic tree analysis showed that 87 samples were European strains, 3 cases were Asian strains, there were no other variations, and G4181A was related to Asian strains. HPV16 L2 gene missense variations G3377A, G3599A, G3703A, and G3757A were significantly more frequent in the case groups than in the control groups.

**Supplementary Information:**

The online version contains supplementary material available at 10.1186/s12920-024-01926-3.

## Introduction

Cervical cancer (CC) is the fourth leading cause of death from malignancy among women, with 604,000 new cases and 342,000 deaths worldwide in 2020 according to the World Health Organization (WHO) [1–3]. The occurrence and development of cervical cancer is related to the economic and health status of the region, and the incidence and mortality rate of cervical cancer in Xinjiang, China, is still very high [4, 5].

Cancer etiology research in the past 25 years has revealed persistent infection of high-risk human papillomavirus (HR-HPV) as a main cause of cervical cancer development and progression [6–8]. The positive rate of HPV in Xinjiang is 14.02% with HPV52, HPV53, HPV16 and HPV18 being the most common types and HPV16 being the most pathogenic among all HPV types [9, 10]. HPV16 is the most dangerous and most preventable virus type. HPV16 is divided into four main variant lineages: A lineage contains EUR (A1-A3) and As (A4); B lineage contains AF-1, C lineage contains (AF-2) and D lineage contains NA (D1), AAII (D2) and AAI (D3). The HPV16 that infects women in Xinjiang, China, are mostly European strains in A lineage [11, 12].

The complete HPV16 genome is approximately 7.9 kb, consisting of six early genes (E1, E2, E4, E5, E6, and E7), two late genes (L1 and L2), and one long control region (LCR) [13]. The HPV16 E4 gene encodes the E4 protein, which is the protein with the highest HPV16 expression. The main amino acids are derived from the E4 ORF, which is contained in the E2 gene [14]. The E4 protein plays a role in viral transmission by enhancing viral replication and the excretion of virions [15, 16]. The HPV16 E5 gene encodes a transmembrane protein with 83 amino acids that serves as an innate immune evasion factor. The transmembrane protein is involved in immune surveillance and immune evasion, leading to persistent viral infection. The transmembrane protein also plays a central role in the regulation of the host immune system, and directly related to the initial stages of cervical cancer development [17, 18]. The HPV L2 gene encodes a minor capsid protein, which promotes retrograde transport of the viral genome, integrating the viral genome into the host gene during the intercellular phase, which may lead to irreversible changes in the cell [19, 20].

Because the HPV16 E4, E5 and L2 genes affect a series of processes of virus invasion, immune escape and transmission, the distribution of the HPV16 E4, E5 and L2 genes in Xinjiang is not yet clear. Therefore, in this study, we focused on the variation in the HPV16 E4, E5 and L2 genes and their distribution in the case group and control group.

## Materials and methods

### Collection of samples

A total of 90 patient samples were collected from Yili Friendship Hospital, Kashgar District People’s Hospital and Shihezi University Affiliated Hospital with HPV16-positive cervical cell samples. All samples had been collected from 2016 to 2017 and patients of age were from 30 to 60 years old. The diagnosis of cervical cancer was confirmed by pathological examination according to “Diagnosis and Treatment, Obstetrics and Gynaecology” and the FIGO stage (International Federation of Gynaecology and Obstetrics, 2009). The inclusion criteria for control groups were HPV16 positive and the absence of lesions or inflammation in the cervix. Informed consent was obtained from all patients. All patients had no history of long-term travel or residence, and samples were collected and stored in a -80 ℃ low-temperature refrigerator. The pathological information of the samples is shown in Table S1 of the supplementary materials.

### HPV genotyping

The HPV genotyping (23 types) was performed with PCR-reverse dot blot hybridization technology (Shenzhen Co., Ltd., China). All of the detection procedures were conducted in accordance with the manufacturer’s instructions [12].

### DNA extraction and PCR amplification of samples

A DNA extraction kit (Tiangen Biochemical Co., Ltd.) was used to extract DNA, which was stored in a -20 °C freezer. Using 1% agarose electrophoresis examined the quality of DNA samples; DNA samples were diluted to a working concentration of 10–20 ng/µL. Samples without DNA bands were re-extracted. The mixed reaction solution (40 µL) consisted of 20 µL of 2×Taq enzyme PCR SuperMix, 1 µL of forward primer (10µmol/L), 1 µL of reverse primer (10µmol/L), 2 µL of DNA sample, and 16 µL of ddH_2_O. The PCR cycling conditions were 94 °C for 5 min; 34 cycles of 94 °C for 30 s, 52 °C for 30 s, 72 °C for 1 min; and 72 °C for 5 min. Using 1% agarose electrophoresis examined the quality of PCR products, and samples with bright and regular bands at 650 bp were qualified for subsequent DNA sequencing. PCR products were stored in a -20 °C freezer. Information on the primers is shown in Table 1.

**Table 1:** Primer information

Primer name	Primer sequences
16B1228E-6 F	5’-GAAGCATCAGTAACTGTGGTAGAGG-3’
16B1228E-6R	5’-TGTAACAATTGCACTTTTATGTTTT-3’
16B1228E-7 F	5’-GCTCACACAAAGGACGGATT-3’
16B1228E-7R	5’-ACGTTTTGTGCGTTTTGCAG-3’
16B1228E-8 F	5’-CATACACATGCACGCTTTTT-3’
16B1228E-8R	5’-TGTTGGAGGCTGCAATACAGA-3’
16B1228E-9 F	5’-GTACCTTCCATTCCCCCAGA-3’
16B1228E-9R	5’-GCACCTATAGATTTTCCACTACGA-3’
16B1228E-10 F	5’-AATATAGCTCCAGATCCTGACTTTT-3’
16B1228E-10R	5’-TGCGTGCAACATATTCATCC-3’

F is the forward primer and R is the reverse primer

### Sequencing

Sequencing was performed by Shanghai Sangon, The Beijing Genomics Institute and other sequencing companies, and the PCR product was purified by SAP (Promega) and EXO I (Epicentre): 0.5 U SAP and 4 U Exo I were added to 8 µl PCR products. The mixture was incubated at 37 °C for 60 min, followed by incubation at 75 °C for 15 min. Finally, the BIG-DYE Terminator V3.1 cycle sequencing kit from ABI Co. was used for sample sequencing on a DNA analyzer (ABI3130XL) after purification with alcohol. The sequencing primers were: 16B1228E-6 F, 16B1228E-7 F, 16B1228E-8 F, 16B1228E-9 F, 16B1228E-10 F (Table 1).

### Phylogenetic analysis of HPV16 variants

The raw sequences were assembled by Molecular Evolutionary Genetics Analysis (MEGA) software and were aligned with the European prototype virus strain (GenBank: NC_001526.4) and the accession numbers of other HPV16 variants including European strains A1-A3: A1 (HQ644283.1, HQ644268.1, HQ644280.1, HQ644282.1), A2: (AF5.36179.1), A3: (HQ644236.1), Asian strains A4 (HQ644235.1, HQ644248.1, AF534061.1, HQ644251.1), African strains B (HQ644238.1, HQ644240.1, HQ644290.1, HQ644298.1) and C (AF472509.1, HQ644239.1, HQ644249.1, HQ644237.1), North American strains: D1 (HQ644257.1), Asian American strains: D2 (HQ644263.1, HQ644277.1, HQ644279.1, HQ644281.1), D3 (HQ644247.1, HQ644255.1, HQ644253.1, AF402678.1). Phylogenetic tree of HPV16 E4, E5, and L2 gene was constructed with MEGA [4, 21], using the Maximum likelihood method, the Bootstrap method (1000 replication), the Tamura 3-parameter model, Gamma distributed with Invariant sites (G + I) and showing only greater than 50% of the Bootstrap values, where the Bootstrap values were > 70% indicates good reliability.

### Statistical analysis

The variation frequency of HPV16 E4, E5, and L2 gene variation was directly counted. SPSS 26.0 software was used to analyze the statistical results and the correlation between HPV16 E4, E5, and L2 single nucleotide variation and cervical cancer. *P* values < 0.05 is accepted as statistically significant in chi-square test.

## Results

### Sequence variation in HPV16 E4, E5 and L2 genes

HPV16 E4, E5 and L2 gene variation results: gene sequencing results of 90 DNA samples with polymorphic sites shown in Tables 2, 3 and 4.

**Table 2:** HPV16 E4 gene variation and amino acid changes

	Variation sites	AA	As	Af	Number of variation samples(*n* = 81)	Variation frequency	Amino acid changes
Synonymous variation	G2504A	G	G	G	1	1.23%	K12K
	T2520C	T	C	T	25	30.86%	L18L
	T2534G	T	T	T	1	1.23%	T22T
	C2546T	T	T	T	76	93.83%	T26T
	C2546A	T	T	T	2	2.47%	T26T
	G2585A	A	A	A	23	28.40%	P39P
	A2603G	A	A	A	1	1.23%	L45L
	T2660C	T	C	T	19	23.46%	C64C
Nonsynonymous variation	T2489G	T	T	T	1	1.23%	C7 W
	G2496T	G	G	G	1	1.23%	A10S
	A2506G	A	A	A	1	1.23%	Y13C
	A2587C	A	A	A	1	1.23%	K40T
	G2596T	G	G	G	1	1.23%	R43I
	G2621T	G	G	G	1	1.23%	Q51H
	G2627T	G	G	G	1	1.23%	Q53H
	G2669C	G	G	G	1	1.23%	E67D
	T2703G	T	T	T	1	1.23%	L79V
	C2712T	C	C	C	1	1.23%	H82Y
	C2716G	C	C	C	1	1.23%	T83R

AA is the Asian–American strain, As is the Asian strain, and Af is the African strain

**Table 3:** HPV16 E5 gene variation and amino acid changes

	Variation sites	AA	As	Af	Number of variation samples(*n* = 90)	Variation frequency	Amino acid changes
Synonymous variation	A2991G	A	A	A	1	1.11%	T2T
	A3018G	A	A	A	1	1.11%	L11L
	G3153A	A	G	G	1	1.11%	A56A
	A3207C	A	A	A	1	1.11%	I74I
	A3213T	A	T	A	20	22.22%	T76T
	A3228G	A	A	A	1	1.11%	L81L
Nonsynonymous variation	C2995T	C	C	C	2	2.22%	L4F
	T3031G	T	T	T	1	1.11%	L16V
	T3068C	T	T	T	1	1.11%	L28S
	A3115C	C	C	C	68	75.56%	I44L
	T3122C	T	T	T	4	4.44%	V46A
	C3127A	G	C	T	10	11.11%	L48I
	C3127G	G	C	T	4	4.44%	L48V
	A3178G	G	G	T	90	100.00%	I65V
	T3179G	T	T	T	1	1.11%	I65R
	A3180G	A	A	A	1	1.11%	I65M
	T3226G	T	T	T	1	1.11%	L81V

AA is the Asian–American strain, As is the Asian strain, and Af is the African strain

**Table 4:** HPV16 L2 gene variation and amino acid changes

	Variation sites	AA	As	Af	Number of variation samples(*n* = 90)	Variation frequency	Amino acid changes
Synonymous variation	A3459C	A	A	A	1	1.11%	P29P
	T3528G	T	T	T	1	1.11%	G52G
	A3531T	A	A	A	1	1.11%	V53V
	T3534C	T	T	T	3	3.33%	F54F
	T3540G	T	T	T	9	10.00%	G56G
	C3573G	G	G	G	1	1.11%	G67G
	A3594T	A	A	A	1	1.11%	P74P
	A3594C	A	A	A	2	2.22%	P74P
	A3627C	A	A	A	1	1.11%	T85T
	G3699T	G	G	G	3	3.33%	V109V
	T3726G	T	T	T	2	2.22%	G118G
	A3735T	C	A	A	1	1.11%	T121T
	T3852C	T	T	T	1	1.11%	N160N
	C3861T	T	T	T	4	4.44%	F163F
	G3879A	G	G	G	1	1.11%	L169L
	A3891C	A	A	A	1	1.11%	T173T
	A3897G	A	A	A	1	1.11%	A175A
	A3906G	A	A	A	1	1.11%	G178G
	T3912C	T	T	T	1	1.11%	H180H
	A3924C	A	A	A	1	1.11%	S184S
	A3972T	A	A	A	1	1.11%	T200T
	C3984T	C	C	C	2	2.22%	S204S
	G4074A	A	A	A	90	100%	Q234Q
	T4083C	T	T	T	1	1.11%	V237V
	T4119C	T	T	T	1	1.11%	L249L
	G4144A	G	G	G	1	1.11%	E258E
	T4275A	T	T	T	4	4.44%	I301I
	T4320G	T	T	T	1	1.11%	G317G
	A4362G	A	C	A	7	7.78%	L330L
	A4437G	A	A	A	1	1.11%	A355A
	A4464G	A	A	A	1	1.11%	G364G
	T4494C	T	T	T	1	1.11%	I374I
	A4518T	A	A	A	1	1.11%	V382V
	A4602G	A	A	A	15	16.67%	L410L
	C4623T	T	C	C	2	2.22%	P417P
	T4626C	T	T	T	1	1.11%	I418I
	A4653G	A	A	A	1	1.11%	L427L
Nonsynonymous variation	C3376G	C	C	C	1	1.11%	R2G
	G3377A	G	G	G	7	7.78%	R2Q
	C3389T	C	C	C	2	2.22%	S6F
	A3396C	A	A	A	1	1.11%	K8N
	G3398C	G	G	G	1	1.11%	R9P
	C3416T	C	C	C	1	1.11%	A15V
	C3424T	C	C	C	1	1.11%	L18F
	G3481A	G	G	G	1	1.11%	E37K
	G3554A	G	G	G	8	8.89%	G61E
	G3578C	C	C	C	5	5.56%	R69P
	A3580C	A	A	A	2	2.22%	T70P
	A3589T	A	A	A	1	1.11%	I73F
	G3599A	G	G	G	7	7.78%	G76E
	G3622A	G	G	G	23	25.56%	D84N
	G3641A	G	G	G	40	44.44%	R90K
	T3656A	T	T	T	2	2.22%	V95E
	G3658A	G	G	G	43	47.78%	D96N
	T3665G	T	T	T	7	7.78%	V98G
	G3688T	G	G	G	1	1.11%	V106F
	T3698G	T	T	T	9	10.00%	V109G
	G3700A	G	G	G	2	2.22%	E110K
	G3703A	G	G	G	32	35.56%	E111K
	G3710T	G	G	G	8	8.89%	S113I
	A3715G	A	A	A	2	2.22%	I115V
	G3757A	G	G	G	27	30.00%	D129N
	A3862T	A	A	A	1	1.11%	T164S
	A3881G	A	A	A	1	1.11%	Q170R
	C3910T	C	C	C	1	1.11%	H180Y
	C3919G	C	C	C	1	1.11%	L183V
	G3952A	G	G	G	2	2.22%	E194K
	G3955A	G	G	G	1	1.11%	E195K
	A3958T	A	A	A	1	1.11%	I196F
	A3976T	A	A	A	1	1.11%	I202F
	G4061T	G	G	G	1	1.11%	R230L
	C4072A	C	C	C	1	1.11%	Q234K
	G4087T	G	G	G	1	1.11%	D239Y
	G4099A	G	G	G	3	3.33%	V243I
	G4129C	G	G	G	1	1.11%	D253H
	T4177G	C	C	C	2	2.22%	S269A
	T4177C	C	C	C	32	35.56%	S269P
	C4178A	C	C	C	1	1.11%	S269Y
	A4180G	A	A	A	2	2.22%	S270G
	G4181A	G	A	G	3	3.33%	S270N
	G4186A	G	G	G	5	5.56%	D272N
	A4190G	A	A	A	1	1.11%	N273S
	A4192C	A	A	A	1	1.11%	S274R
	A4203G	A	A	A	1	1.11%	I277M
	C4207A	C	C	C	1	1.11%	P279T
	A4288C	A	A	A	1	1.11%	I306L
	A4301G	A	A	A	1	1.11%	Q310R
	A4318G	A	A	A	1	1.11%	S316G
	G4322A	G	G	G	1	1.11%	G317E
	T4327G	T	T	T	1	1.11%	S319A
	A4339G	A	A	A	1	1.11%	K323E
	A4362C	A	C	A	46	51.11%	L330F
	A4362T	A	C	A	35	38.89%	L330F
	C4367A	C	C	C	1	1.11%	T332N
	G4372A	A	G	A	3	3.33%	D334N
	A4385G	A	A	A	1	1.11%	E338G
	G4390A	G	G	G	1	1.11%	E340K
	A4405C	A	A	A	1	1.11%	T345P
	T4441A	T	T	T	1	1.11%	S357T
	A4460C	A	A	A	1	1.11%	N363T
	C4505T	T	T	C	6	6.67%	S378F
	A4628C	A	A	A	4	4.44%	N419T
	A4630G	A	A	A	1	1.11%	I420V
	A4654C	A	C	A	21	23.33%	I428L

AA is the Asian–American strain, As is the Asian strain, and Af is the African strain

Among the 90 HPV16-positive samples, 81 samples had HPV16 E4 gene sequence variations in 18 nucleotide sites with 8 synonymous and 11 missense. The common synonymous variations were nt2520 (T-C) (25/81, 30.86%), nt2546 (C-T/A) (76/81, 93.83%; 2/81, 2.47%), nt2585 (G-A) (23/81, 28.40%), and nt2660 (T-C) (19/81, 23.46%). The number of variation of missense variation sites did not exceed 1. The frequency of missense variation was much lower than that of synonymous variations, indicating that the E4 gene was relatively conserved and had a stabilizing effect on the spread of the virus.

All of the 90 HPV16-positive samples had HPV16 E5 sequence variations in 16 sites with 6 synonymous and 11 missense variations. As shown in Table 3, the sites with high synonymous variation in the E5 gene were nt3213 (A-T) (20/90, 22.22%); the sites with more than 1 missense variation were: C2995T, A3115C, T3122C, C3127A/G, and A3178G, leading to amino acid changes leucine to phenylalanine (L4F), isoleucine to leucine (I44L), valine to alanine (V46A), leucine to isoleucine/valine (L48I/V) and isoleucine to valine (I65V) respectively. The A3115C and A3178G variations had very high frequency, 75.56% and 100%, respectively, and the simultaneous occurance of these two variations may significantly change the structure of the E5 protein and indirectly change the ability of virus immune escape.

Among the 90 HPV16 positive samples, all samples had L2 gene sequence variation in 100 nucleotide sites with 37 synonymous and 67 missense variations. The sites with high synonymous variations were nt4074 (G-A) (90/90, 100%) and nt4602 (A-G) (15/90, 16.67%) (Table 4). The most common missense variations were G3622A, G3641A, G3658A, G3703A, G3757A, T4177C, A4362C/T and A4654C, which led the amino acid changes aspartic acid to asparagine (D84N), arginine to lysine (R90K), aspartic acid to asparagine (D96N), glutamic acid to lysine (E111K), aspartic acid to asparagine (D129N), serine to proline (S269P), leucine to phenylalanine (L330F), and isoleucine to leucine (I428L). Through the three gene variation sites of HPV16 E4, E5 and L2, it can be found that the frequency of missense variation of the E4 gene is lower than that of the E5 and L2 genes, indicating that the E4 gene is more conserved than these two genes.

### Phylogenetic tree analysis of the nucleotide sequences of HPV16 E4, E5 and L2

**Fig. 1:**
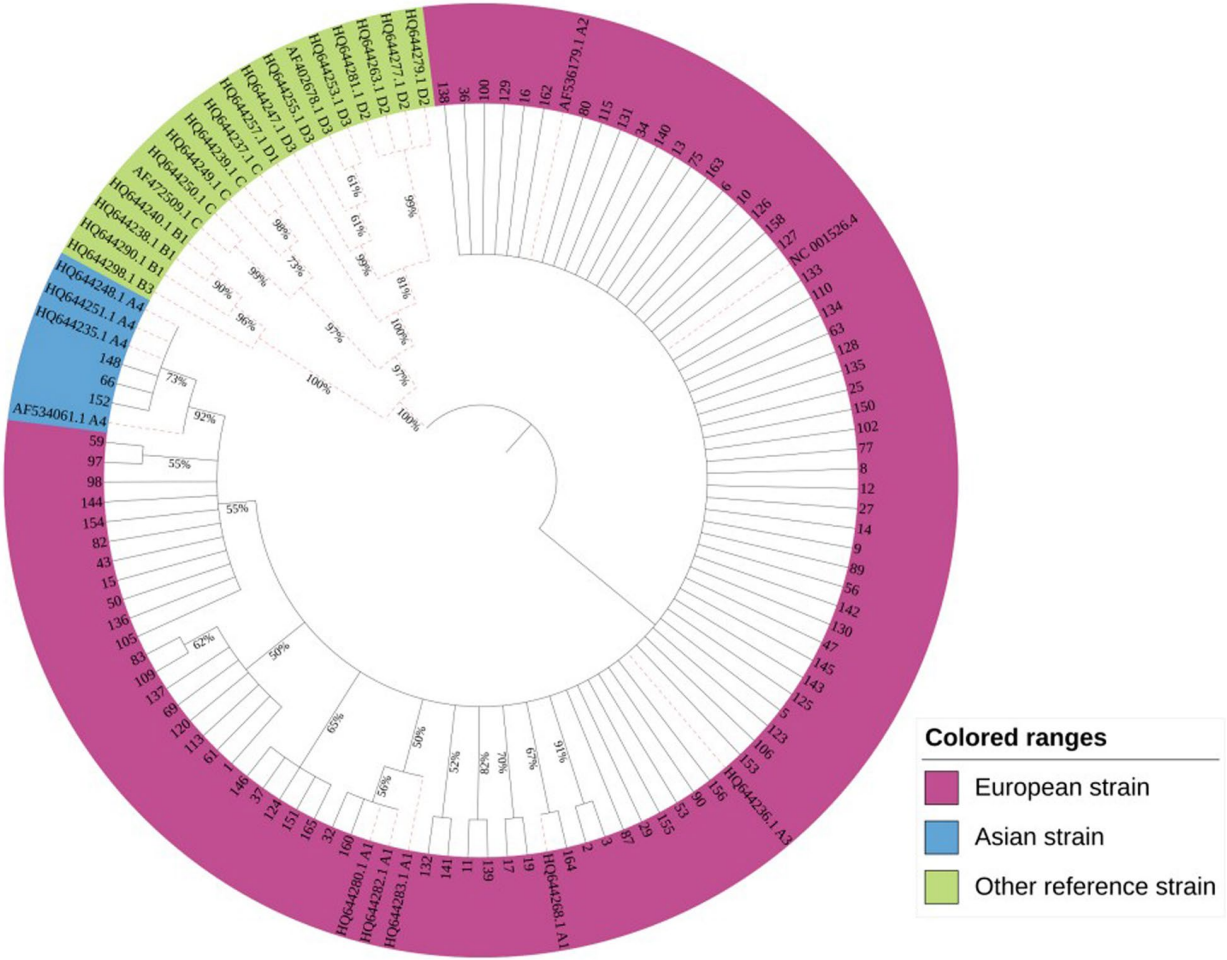
Phylogenetic tree analysis of HPV16 E4 E5 and L2 genes, the red dotted line is the reference virus strain

The Maximum likelihood method phylogenetic tree constructed with HPV16 E4, E5 and L2 gene sequences showed that 87 of the 90 HPV16 positive samples were European strains and 3 samples were Asian strains. No African, American or Asian-American strains were found. The Asian strains were associated with the missense variant G4181A, and the phylogenetic tree was shown in Fig. 1.

### Genetic variation of genomic HPV16 E4, E5 and L2 in the case and control groups

#### Genetic variation of genomic HPV16 E4 in the case and control groups

The pathological information of the samples was statistical, including 47 cases in the control groups and 43 cases in the case groups. There were 6 synonymous variations and 1 missense variations in the control groups (non-cervical cancer group) (Table 5). In comparison, there were 7 synonymous variations and 10 missense variations in the case groups (cervical cancer group). The sequence variations did not differ significantly between the control groups and the case groups (*P* > 0.05) (Table 5). Most of the missense variations appeared in the case group, indicating the trend of E4 gene missense variations in the case group.

**Table 5:** Genetic variation of genomic HPV16 E4 in case and control groups

	Variation sites	Control groups		Case groups		*P* values	Amino acid changes
		Number of variation samples(*n* = 47)	Variation frequency	Number of variation samples(*n* = 43)	Variation frequency		
Synonymous variation	G2504A	1	2.13%	0	0.00%	0.336	K12K
	T2520C	13	27.66%	12	27.91%	0.979	L18L
	T2534G	0	0.00%	1	2.33%	0.293	T22T
	C2546T	40	85.11%	36	83.72%	0.856	T26T
	C2546A	1	2.13%	1	2.33%	0.949	T26T
	G2585A	11	23.40%	12	27.91%	0.625	P39P
	A2603G	0	0.00%	1	2.33%	0.293	L45L
	T2660C	9	19.15%	10	23.26%	0.633	C64C
Nonsynonymous variation	T2489G	0	0.00%	1	2.33%	0.293	C7 W
	G2496T	0	0.00%	1	2.33%	0.293	A10S
	A2506G	0	0.00%	1	2.33%	0.293	Y13C
	A2587C	0	0.00%	1	2.33%	0.293	K40T
	G2596T	0	0.00%	1	2.33%	0.293	R43I
	G2621T	0	0.00%	1	2.33%	0.293	Q51H
	G2627T	0	0.00%	1	2.33%	0.293	Q53H
	G2669C	1	2.13%	0	0.00%	0.336	E67D
	T2703G	0	0.00%	1	2.33%	0.293	L79V
	C2712T	0	0.00%	1	2.33%	0.293	H82Y
	C2716G	0	0.00%	1	2.33%	0.293	T83R

The HPV16 E4 reference sequence is NC_001526.4

#### Genetic variation of genomic HPV16 E5 in the case and control groups

The control groups (non-cervical cancer group) had 3 synonymous variations and 8 missense variations. In comparison, the case groups (cervical cancer group) had 4 synonymous variations and 8 missense variations (Table 6). The statistical results showed that the synonymous variations A3213T was significantly higher than the control groups in the case groups, and the difference was statistically significant (*P* = 0.024). It is worth mentioning that the variation frequency of the missense variation A3115C in the case groups was 83.72%, which was higher than the frequency of variation in the control groups (68.09%), but the difference was not statistically significant (*P* > 0.05).

**Table 6:** Genetic variation of genomic HPV16 E5 in case and control groups

	Variation sites	Control groups		Case groups		*P* values	Amino acid changes
		Number of variation samples(*n* = 47)	Variation frequency	Number of variation samples(*n* = 43)	Variation frequency		
Synonymous variation	A2991G	0	0.00%	1	2.33%	0.293	T2T
	A3018G	1	2.13%	0	0.00%	0.336	L11L
	G3153A	0	0.00%	1	2.33%	0.293	A56A
	A3207C	1	2.13%	0	0.00%	0.336	I74I
	A3213T^a^	6	12.77%	14	32.56%	0.024	T76T^a^
	A3228G	0	0.00%	1	2.33%	0.293	L81L
Nonsynonymous variation	C2995T	0	0.00%	2	4.65%	0.135	L4F
	T3031G	1	2.13%	0	0.00%	0.336	L16V
	T3068C	1	2.13%	0	0.00%	0.336	L28S
	A3115C	32	68.09%	36	83.72%	0.085	I44L
	T3122C	3	6.38%	1	2.33%	0.351	V46A
	C3127A	6	12.77%	4	9.30%	0.601	L48I
	C3127G	1	2.13%	3	6.98%	0.265	L48V
	A3178G	47	100.00%	43	100.00%	1	I65V
	T3179G	1	2.13%	0	0.00%	0.336	I65R
	A3180G	0	0.00%	1	2.33%	0.293	I65M
	T3226G	0	0.00%	1	2.33%	0.293	L81V

The HPV16 E5 reference sequence is NC_001526.4

^a^Represents sites with significant differences

#### Genetic variation of genomic HPV16 L2 in the case and control groups

There were 24 synonymous variations and 44 missense variations in the control groups and 22 synonymous variations and 44 missense variations in the case groups (Table 7), among which the missense variations were G3377A (*P* = 0.036), G3599A (*P* = 0.004), G3703A (*P* = 0.038), and G3757A (*P* = 0.019). The frequency of variations in the case groups was significantly higher than that in the control groups, and the difference was statistically significant (*P* < 0.05). The amino acid changes were arginine to glutamine (R2Q), alanine to glutamic acid (G76E), glutamate to lysine (E111K), and aspartate to asparagine (D129N).

**Table 7:** Genetic variation of genomic HPV16 L2 in case and control groups

	Variation sites	Control groups		Case groups		*P* values	Amino acid changes
		Number of variation samples(*n* = 47)	Variation frequency	Number of variation samples(*n* = 43)	Variation frequency		
Synonymous variation	A3459C	0	0.00%	1	2.33%	0.293	P29P
	T3528G	0	0.00%	1	2.33%	0.293	G52G
	A3531T	0	0.00%	1	2.33%	0.293	V53V
	T3534C	3	6.38%	0	0.00%	0.092	F54F
	T3540G	2	4.26%	7	16.28%	0.058	G56G
	C3573G	0	0.00%	1	2.33%	0.293	G67G
	A3594T	0	0.00%	1	2.33%	0.293	P74P
	A3594C	1	2.13%	1	2.33%	0.949	P74P
	A3627C	0	0.00%	1	2.33%	0.293	T85T
	G3699T	1	2.13%	2	4.65%	0.505	V109V
	T3726G	0	0.00%	2	4.65%	0.135	G118G
	A3735T	0	0.00%	1	2.33%	0.293	T121T
	T3852C	0	0.00%	1	2.33%	0.293	N160N
	C3861T	3	6.38%	1	2.33%	0.351	F163F
	G3879A	1	2.13%	0	0.00%	0.336	L169L
	A3891C	1	2.13%	0	0.00%	0.336	T173T
	A3897G	1	2.13%	0	0.00%	0.336	A175A
	A3906G	1	2.13%	0	0.00%	0.336	G178G
	T3912C	0	0.00%	1	2.33%	0.293	H180H
	A3924C	1	2.13%	0	0.00%	0.336	S184S
	A3972T	1	2.13%	0	0.00%	0.336	T200T
	C3984T	2	4.26%	0	0.00%	0.171	S204S
	G4074A	47	100.00%	43	100%	1	Q234Q
	T4083C	1	2.13%	0	0.00%	0.336	V237V
	T4119C	1	2.13%	0	0.00%	0.336	L249L
	G4144A	1	2.13%	0	0.00%	0.336	E258E
	T4275A	1	2.13%	3	6.98%	0.265	I301I
	T4320G	0	0.00%	1	2.33%	0.293	G317G
	A4362G	5	10.64%	2	4.65%	0.289	L330L
	A4437G	1	2.13%	0	0.00%	0.336	A355A
	A4464G	1	2.13%	0	0.00%	0.336	G364G
	T4494C	1	2.13%	0	0.00%	0.336	I374I
	A4518T	1	2.13%	0	0.00%	0.336	V382V
	A4602G	6	12.77%	9	20.93%	0.299	L410L
	C4623T	1	2.13%	1	2.33%	0.949	P417P
	T4626C	0	0.00%	1	2.33%	0.293	I418I
	A4653G	0	0.00%	1	2.33%	0.293	L427L
Nonsynonymous variation	C3376G	0	0.00%	1	2.33%	0.293	R2G
	G3377A^a^	1	2.13%	6	13.95%	0.036	R2Q^a^
	C3389T	0	0.00%	2	4.65%	0.135	S6F
	A3396C	1	2.13%	0	0.00%	0.336	K8N
	G3398C	0	0.00%	1	2.33%	0.293	R9P
	C3416T	0	0.00%	1	2.33%	0.293	A15V
	C3424T	0	0.00%	1	2.33%	0.293	L18F
	G3481A	0	0.00%	1	2.33%	0.293	E37K
	G3554A	2	4.26%	6	13.95%	0.106	G61E
	G3578C	1	2.13%	4	9.30%	0.138	R69P
	A3580C	0	0.00%	2	4.65%	0.135	T70P
	A3589T	0	0.00%	1	2.33%	0.293	I73F
	G3599A^a^	0	0.00%	7	16.28%	0.004	G76E^a^
	G3622A	9	19.15%	14	32.56%	0.145	D84N
	G3641A	18	38.30%	22	51.16%	0.22	R90K
	T3656A	0	0.00%	2	4.65%	0.135	V95E
	G3658A	21	44.68%	22	51.16%	0.539	D96N
	T3665G	2	4.26%	5	11.63%	0.192	V98G
	G3688T	0	0.00%	1	2.33%	0.293	V106F
	T3698G	3	6.38%	6	13.95%	0.232	V109G
	G3700A	0	0.00%	2	4.65%	0.135	E110K
	G3703A^a^	12	25.53%	20	46.51%	0.038	E111K^a^
	G3710T	2	4.26%	6	13.95%	0.106	S113I
	A3715G	0	0.00%	2	4.65%	0.135	I115V
	G3757A^a^	9	19.15%	18	41.86%	0.019	D129N^a^
	A3862T	1	2.13%	0	0.00%	0.336	T164S
	A3881G	1	2.13%	0	0.00%	0.336	Q170R
	C3910T	1	2.13%	0	0.00%	0.336	H180Y
	C3919G	1	2.13%	0	0.00%	0.336	L183V
	G3952A	2	4.26%	0	0.00%	0.171	E194K
	G3955A	1	2.13%	0	0.00%	0.336	E195K
	A3958T	1	2.13%	0	0.00%	0.336	I196F
	A3976T	1	2.13%	0	0.00%	0.336	I202F
	G4061T	1	2.13%	0	0.00%	0.336	R230L
	C4072A	1	2.13%	0	0.00%	0.336	Q234K
	G4087T	1	2.13%	0	0.00%	0.336	D239Y
	G4099A	2	4.26%	1	2.33%	0.61	V243I
	G4129C	1	2.13%	0	0.00%	0.336	D253H
	T4177G	2	4.26%	0	0.00%	0.171	S269A
	T4177C	17	36.17%	15	34.88%	0.899	S269P
	C4178A	1	2.13%	0	0.00%	0.336	S269Y
	A4180G	2	4.26%	0	0.00%	0.171	S270G
	G4181A	2	4.26%	1	2.33%	0.61	S270N
	G4186A	3	6.38%	2	4.65%	0.72	D272N
	A4190G	0	0.00%	1	2.33%	0.293	N273S
	A4192C	1	2.13%	0	0.00%	0.336	S274R
	A4203G	1	2.13%	0	0.00%	0.336	I277M
	C4207A	0	0.00%	1	2.33%	0.293	P279T
	A4288C	0	0.00%	1	2.33%	0.293	I306L
	A4301G	0	0.00%	1	2.33%	0.293	Q310R
	A4318G	0	0.00%	1	2.33%	0.293	S316G
	G4322A	1	2.13%	0	0.00%	0.336	G317E
	T4327G	0	0.00%	1	2.33%	0.293	S319A
	A4339G	0	0.00%	1	2.33%	0.293	K323E
	A4362C	25	53.19%	21	48.84%	0.68	L330F
	A4362T	16	34.04%	19	44.19%	0.324	L330F
	C4367A	1	2.13%	0	0.00%	0.336	T332N
	G4372A	2	4.26%	1	2.33%	0.61	D334N
	A4385G	0	0.00%	1	2.33%	0.293	E338G
	G4390A	1	2.13%	0	0.00%	0.336	E340K
	A4405C	0	0.00%	1	2.33%	0.293	T345P
	T4441A	1	2.13%	0	0.00%	0.336	S357T
	A4460C	0	0.00%	1	2.33%	0.293	N363T
	C4505T	4	8.51%	2	4.65%	0.463	S378F
	A4628C	2	4.26%	2	4.65%	0.929	N419T
	A4630G	1	2.13%	0	0.00%	0.336	I420V
	A4654C	8	17.02%	12	27.91%	0.215	I428L

The HPV16 L2 reference sequence is NC_001526.4

^a^Represents sites with significant differences

## Discussion

Most of the research on HPV gene variation has focused on the E6 and E7 genes, while there are fewer studies on E4, E5, and L2 gene variations, and the understanding of HPV16 E4, E5 and L2 gene variations in Xinjiang is even more insufficient. The HPV16 E4, E5 and L2 genes dominate virion propagation, immune surveillance and escape, and the integration of the viral genome into the chromosomes of the nucleus [15, 18]; Sequence variation in E4, E5 and L2 genes, thus, will directly affect the ability of HPV virus to invade, immune, and spread.

We found that 87 cases (87/90, 96.67%) of the 90 HPV16 positive samples in Xinjiang were European strains, and the other 3 cases (3/90, 3.33%) were Asian strains. We speculated that the Asian strain was directly related to the L2 gene missense variation G4181A through bioinformatics comparison. The literature reported that the amino acid variations of HPV16 E4 protein include T22A, P36T, A43K, Q53R, L62I and L62P, which of these amino acid variations are related to the severity of cervical malignancy [22]. The E4 gene also encodes the E1^E4 protein, the first five amino acids of which are derived from E1 ORF, while the remaining amino acids are derived from E4 ORF. nine amino acid variations (A7V, A7P, L16I, D45E, L59I, L59T, Q66P, S72F, H75Q) were detected in the E1^E4 protein, and these were associated with the severity of cervical malignancy [23]. In this study, it was found that the amino acid variation of E4 protein (E1^E4 protein) : C7W, A10S (A7S), Y13C (Y10C), K40T (K37T), R43I (R40I), Q51H (Q48H), Q53H (Q50H), E67D (E64D), L79V (L76V), H82Y (H79Y) and T83R (T80R), which of amino acid variations had low frequency. Therefore, since the amino acid variations the frequency of E4 protein were much lower than that of E5 and L2 proteins, indicating that E4 gene was more conserved than E5 and L2 genes. Moreover, the missense variations of the E4 gene that was new variations were concentrated in the case group (cervical cancer). The HPV16 E4 gene is located in the central position of the E2 gene, which encodes the hinge domain in the E2 protein. The most common synonymous variations in the E4 gene, T2520C (30.86%), C2546T (93.83%) and G2585A (28.40%), are also missense variations in the hinge region of the E2 gene, which cause amino acid changes in the E2 protein and affect the viral replication process [14]. The reported that amino acid variations of E5 protein include F19I, V21A, C24S, L27P, P31L, I44L, L47S, L48A, V62A, I65V, I65L, and L73V [22, 24]. We found that the new amino acid variations of E5 protein include L4F, L16V, L28S, V46A, L48I, L48V, I65R, I65M and L81V, which L48I and L48V were the amino acid variations with high frequency. It was reported that a total of 17 amino acid variations with high frequency in L2 proteins, including D43E, S122P, V243I, T245A, L266F, L266V, S269P, L330F, D334N, T351P, T351S, T352P, T352A, S378VS378F, S384A, V385I, I420T, A424T, I428L and A443G. The amino acid variation I428L was present almost uniquely in Asia, and the frequency of S269P and L330F was higher than that of the reference amino acid, at position 330 phenylalanine of L2 protein was more common than the reference amino acid leucine in Europe, Asia, and North America [25]. We also found high-frequency variations of S269P and L330F in the L2 protein, which were consistent with the previous reports. In addition, the other high frequency amino acid variations have also been found in L2 protein, including D84N, R90K, D96N, E111K and D129N (Table 4). We found that L2 missense variations G3377A (R2Q), G3599A (G76E), G3703A (E111K), and G3757A (D129N), were in significantly higher frequency in the case groups (cervical cancer) than in the control groups(non-cervical cancer) (*P* < 0.05). These variations may affect the integration of HPV16 viral genome into the cell chromosomes.

Xinjiang is a multiethnic region. We included a total of 52 Han ethnic group samples in the 90 samples we studied, including 31 samples in the control groups and 21 samples in the case groups, and recounted several loci with more variations (see Table 8 for details). We found that the missense variation A3115C of the E5 gene was in significantly higher frequency in the case groups than in the control groups (*P* = 0.02), and the difference was statistically significant. However, as seen in Table 6 above, A3115C did not differ between the case and control groups in the 90 samples, so the A3115C variation may have different effects on different ethnic groups, which needs further validation.

**Table 8:** Distribution of high variation frequency sites in Han nationality

Variation sites	Control groups		Case groups		*P* values	Amino acid changes
	Number of variation samples(*n* = 31)	Variation frequency	Number of variation samples(*n* = 21)	Variation frequency		
T2520C	11	35.48%	8	38.10%	0.848	L18L
C2546T	26	83.87%	19	90.48%	0.494	T26T
G2585A	9	29.03%	9	42.86%	0.304	P39P
A3115C^a^	19	61.29%	19	90.48%	0.02	I44L^a^
C3127A	4	12.90%	1	4.76%	0.329	L48I
C3127G	1	3.23%	2	9.52%	0.339	L48V
G3622A	3	9.68%	6	28.57%	0.77	D84N
G3641A	10	32.26%	10	47.62%	0.264	R90K
G3658A	11	35.48%	11	52.38%	0.226	D96N
T4177C	13	41.94%	11	52.38%	0.458	S269P
A4362C	21	67.74%	11	52.38%	0.264	L330F
A4362T	7	22.58%	7	33.33%	0.391	L330F
A4654C	8	25.81%	8	38.10%	0.346	I428L

^a^Represents sites with significant differences

The current study revelaed for the first time sequence variations in HPV16 E4, E5 and L2 genes and in Xinjiang, and the distribution of these variations among different ethnic groups. The sample size of the current study is relatively small and should be increased in future studies, in particular to include samples from more ethnic groups. Based on findings from the current study, variations A3115C, G3377A, G3599A, G3703A and G3757A should be further investigated in cell experiments to determine whether they affect the viral immunity and the integration of viral genome in cell chromosomes.

## Conclusion

Phylogenetic tree analysis showed that 87 samples were European strains, 3 cases were Asian strains, there were no other variants, and G4181A was related to Asian strains. HPV16 L2 gene missense variants G3377A, G3599A, G3703A, and G3757A were significantly more frequent in the case groups than in the control groups.

### Supplementary Information

Supplementary Material 1.

## Data Availability

No datasets were generated or analysed during the current study.
