# Supplementary material for: Analysis of human papillomavirus type 16 E4, E5 and L2 gene variations among women with cervical infection in Xinjiang, China
Source: BMC Med Genomics. 2024 Jul 4;17:179. doi: 10.1186/s12920-024-01926-3 (PMC11225290; doi:10.1186/s12920-024-01926-3)
Supplement: Supplementary file 1 — Supplementary Material 1. [file 12920_2024_1926_MOESM1_ESM.docx]

Table S1 Sample pathological information

| serial number | Sample number | Disease cases | Pathological information |
| --- | --- | --- | --- |
| 1 | 5 | 74**01 | Cervicitis |
| 2 | 6 | 74**11 | Cervicitis |
| 3 | 8 | 6542**588 | Cervicitis |
| 4 | 10 | 65420**833 | Cervicitis |
| 5 | 11 | 74**64 | Cervicitis |
| 6 | 12 | 74**57 | Cervicitis |
| 7 | 15 | 74**85 | Normal cervix |
| 8 | 16 | 74**44 | Normal cervix |
| 9 | 17 | 66**44 | Cervicitis |
| 10 | 25 | 75**97 | Normal cervix |
| 11 | 32 | 74**57 | Cervicitis |
| 12 | 34 | 654**5824 | Normal cervix |
| 13 | 36 | 75**27 | Cervicitis |
| 14 | 63 | 6542**9472 | Normal cervix |
| 15 | 66 | 75**02 | CIN1 |
| 16 | 77 | 71**97 | Normal cervix |
| 17 | 80 | 76**85 | Cervicitis |
| 18 | 87 | 76**15 | Cervicitis |
| 19 | 89 | 76**68 | Cervicitis |
| 20 | 90 | 6542**329 | Normal cervix |
| 21 | 102 | 76**90 | Cervicitis |
| 22 | 105 | 76**11 | Normal cervix |
| 23 | 110 | 76**42 | Normal cervix |
| 24 | 120 | 76**07 | Normal cervix |
| 25 | 127 | 6542**299 | Normal cervix |
| 26 | 128 | 6542**507 | Cervicitis |
| 27 | 129 | 654**956 | Cervicitis |
| 28 | 130 | 654**841 | Cervicitis |
| 29 | 131 | 65420**116 | Normal cervix |
| 30 | 133 | 77**21 | Cervicitis |
| 31 | 134 | 6542**856 | Cervicitis |
| 32 | 135 | 6542**093 | Cervicitis |
| 33 | 138 | 74**00 | Normal cervix |
| 34 | 139 | 6542**590 | Normal cervix |
| 35 | 140 | 6542**626 | Cervicitis |
| 36 | 141 | 6542**391 | Normal cervix |
| 37 | 144 | 78**59 | Normal cervix |
| 38 | 146 | 78**09 | Normal cervix |
| 39 | 148 | 6542**591 | Normal cervix |
| 40 | 151 | 6542**406 | Normal cervix |
| 41 | 153 | 6542**680 | Normal cervix |
| 42 | 154 | 6542**478 | Normal cervix |
| 43 | 155 | 6542**108 | Cervicitis |
| 44 | 156 | 79**77 | Cervicitis |
| 45 | 158 | 6542**998 | Normal cervix |
| 46 | 162 | 6542**863 | Normal cervix |
| 47 | 164 | 6542**991 | Normal cervix |
| 48 | 1 | 74**67 | Cervical cancer |
| 49 | 2 | 74**90 | Cervical cancer |
| 50 | 3 | 74**11 | Cervical cancer |
| 51 | 9 | 74**98 | Cervical cancer |
| 52 | 13 | 71**91 | Cervical cancer |
| 53 | 14 | 6542**513 | Cervical cancer |
| 54 | 19 | 74**71 | Cervical cancer |
| 55 | 27 | 75**70 | Cervical cancer |
| 56 | 29 | 75**55 | Cervical cancer |
| 57 | 37 | 754225 | Cervical cancer |
| 58 | 43 | 6542**860 | Cervical cancer |
| 59 | 47 | 74**23 | Cervical cancer |
| 60 | 50 | 6542**302 | Cervical cancer |
| 61 | 53 | 6542**335 | Cervical cancer |
| 62 | 56 | 75**38 | Cervical cancer |
| 63 | 59 | 6542**664 | Cervical cancer |
| 64 | 61 | 74**67 | Cervical cancer |
| 65 | 75 | 6542**267 | Cervical cancer |
| 66 | 82 | 6542**161 | Cervical cancer |
| 67 | 83 | 6542**078 | Cervical cancer |
| 68 | 97 | 76**46 | Cervical cancer |
| 69 | 98 | 76**71 | Cervical cancer |
| 70 | 100 | 6542**130 | Cervical cancer |
| 71 | 106 | 57**93 | Cervical cancer |
| 72 | 109 | 6542**894 | Cervical cancer |
| 73 | 113 | 6542**845 | Cervical cancer |
| 74 | 115 | 54**17 | Cervical cancer |
| 75 | 123 | 76**75 | Cervical cancer |
| 76 | 124 | 6542**517 | Cervical cancer |
| 77 | 125 | 6542**795 | Cervical cancer |
| 78 | 126 | 6542**418 | Cervical cancer |
| 79 | 132 | 6542**548 | Cervical cancer |
| 80 | 136 | 6542**675 | Cervical cancer |
| 81 | 137 | 6542**481 | Cervical cancer |
| 82 | 142 | 6542**449 | Cervical cancer |
| 83 | 143 | 6542**078 | Cervical cancer |
| 84 | 145 | 6542**941 | Cervical cancer |
| 85 | 150 | 78**81 | Cervical cancer |
| 86 | 152 | 6542**838 | Cervical cancer |
| 87 | 160 | 64**15 | Cervical cancer |
| 88 | 163 | 78**68 | Cervical cancer |
| 89 | 165 | 76**84 | Cervical cancer |
| 90 | 69 | 75**72 | CIN3 |
